# Involvement of Neuropeptide Y within Paraventricular Nucleus in Electroacupuncture Inhibiting Sympathetic Activities in Hypertensive Rats

**DOI:** 10.1155/2022/9990854

**Published:** 2022-01-18

**Authors:** Qi Zhang, Yingying Tan, Xin Wen, Fangfang Li

**Affiliations:** ^1^Shaanxi Key Laboratory of Chinese Medicine Encephalopathy, Shaanxi University of Chinese Medicine, Xianyang, China; ^2^School of Basic Medical Science, Shaanxi University of Chinese Medicine, Xianyang, China

## Abstract

Although electroacupuncture (EA) has been used to decrease the blood pressure (BP) clinically, the underlying mechanisms are not clearly clarified. This study aimed to assess the hypothesis that EA treatment exerts a hypotensive action via suppressing sympathetic activities and modulating neuropeptide Y (NPY) function within the paraventricular nucleus (PVN) of hypertensive rats. Male Sprague-Dawley rats were selected for the experiment, and the hypertensive models were established by the two-kidney, one-clip (2K1C) method. Then, the rats were randomly assigned to the sham group, 2K1C group, 2K1C plus EA group, and 2K1C plus sham EA group. EA treatment at the acupoints ST36 and ST40 overlying the peroneal nerves was given once a day for 30 days. The radiotelemetry system was applied to collect the arterial BP recordings. Power spectral analyses of BP variability, BP responses to ganglionic blockade, and plasma levels of norepinephrine and epinephrine were performed to assess the changes in sympathetic nerve activity. Real-time PCR and Western blots were carried out to examine the expression of NPY system in the PVN. The responses of PVN microinjection with NPY Y1R antagonist BIBO3304 were detected to check the endogenous NPY tone. The results showed that the enhanced arterial BP and sympathetic activities were effectively reduced by 30 days of EA treatment, and baroreflex sensitivity was improved in 2K1C hypertensive rats. The level of NPY mRNA and protein expression in the PVN was markedly upregulated by EA treatment in 2K1C rats. In addition, the pressor responses of PVN microinjection with NPY Y1R antagonist BIBO3304 in 2K1C models were remarkably augmented by the EA stimulation. Our results indicate that the increased NPY expression and function in the PVN induced by EA treatment contribute to antihypertensive and sympathetic suppression on hypertensive rats. The findings may elucidate the underlying mechanisms of the acupuncture to be a potential therapeutic strategy against hypertension.

## 1. Introduction

In Asia, acupuncture has been used as a complementary and alternative medical therapeutic method for over 2,000 years. Even nowadays, acupuncture is widely practiced for treating various disorders in many countries, especially in China [1]. Electroacupuncture (EA) is an adjusted therapeutic method compared with the traditional acupuncture, in which the needle stimulation is applied by a pulsating electrical current to the acupoints [2]. The therapeutic and regulatory effects of traditional acupuncture or EA have been confirmed by numerous researches, including clinical and animal experiments, on some types of hypertensions [3–5]. Based on the studies, the nervous system, neurotransmitters, and endogenous substances are suggested to be involved in this effect of acupuncture [6]. However, the underlying neural mechanisms related to the antihypertension effects of traditional acupuncture or EA treatment remain to be further clarified. Elevated sympathetic activity is a hallmark of development of essential hypertension [7]. There are various interventions in the central nervous system such as interruption of neuromodulators in specific sympathetic regulation areas and reverse hypertension [8, 9]. The paraventricular nucleus (PVN), which is located in the hypothalamus, is considered as a key area that integrates sympathetic outflow via both direct and indirect projections to sympathetic preganglionic neurons [10]. Studies in both humans and animals have shown that traditional acupuncture or EA exhibits the abilities to inhibit sympathetic activity and sympathoexcitatory reflex responses [11–14]. Thus, influencing the PVN neuronal activity is probably one of the key mechanisms of EA on regulating sympathetic outflow in hypertension.

Neuropeptide Y (NPY) is a kind of polypeptide with 36 amino acids, which is found abundantly in the brain. NPY has been implicated in sympathoinhibitory and orexigenic effects [15, 16]. These actions are mediated via Y1, Y2, and Y5 receptors, which are distributed throughout the hypothalamus and brainstem [17]. The PVN is the main area where the NPY directly suppresses presympathetic neurons projecting to the rostral ventrolateral medulla, which initiates basal sympathetic tone [18]. In addition, PVN is densely innervated by NPY-containing axons originating from arcuate nucleus, which form functional inhibitory synapses on PVN neurons [19]. However, it is unknown if NPY in the PVN contributes to acupuncture's modulation of sympathetic outflow.

We conducted the study to test the hypothesis that the reducing effects on hypertension and sympathetic activity caused by EA stimulation may associate with modulating NPY activity within the PVN of hypertensive rats.

## 2. Materials and Methods

### 2.1. Animals

Male Sprague-Dawley (SD) rats (wt. 200–220 g) were selected for the experiment. The SD rats were purchased from the Laboratory Animal Center of Xi'an Jiaotong University and fed in a room with 22 ± 2°C temperature, 50 ± 5% humidity, and 12 h : 12 h light-dark cycle with free access to rat chow and tap water. All experiments were conducted in accordance with the Guide for the Care and Use of Laboratory Animals (NIH publication, eighth edition, 2011) and approved by the Institutional Animal Care and Use Committee of Shaanxi University of Chinese Medicine.

### 2.2. Radiotelemetry

SD rats were implanted with a radiotelemetry system for conscious BP measurements as reported previously [20]. Rats were briefly anesthetized with 3% isoflurane inhalation. Under sterile condition, the peritoneal cavity was exposed and the abdominal aorta was carefully separated. A catheter of telemetry BP probe (model TA11PA-C40, Data Sciences International, St. Paul, MN, USA) was inserted into the lower abdominal aorta, and the transmitter body was secured to the ventral abdominal muscle. After the rats recovered for 1 week, Dataquest IV system (version 4.33, Data Sciences International, Saint Paul, MN, United States) was used to process and record the telemetry signals.

### 2.3. Experimental Grouping and Two-Kidney, One-Clip (2K1C) Hypertension Modeling

After the radiotelemetry system implantation, rats were randomly assigned to one of the following groups: sham group (*n* = 15), 2K1C hypertensive group (*n* = 15), 2K1C plus EA group (2K1C + EA, *n* = 15), and 2K1C plus sham EA group (2K1C + SEA, *n* = 15).

The 2K1C hypertension model was produced following our previous report [20, 21]. In simple terms, the rats were anesthetized with pentobarbital (i.p., 40 mg/kg), followed by exposing the right kidney via a retroperitoneal flank incision under sterile surgical condition. For 2K1C modeling, a silver clip (0.20 mm diameter) was placed in the right renal artery. Sham-operated rats performed the same surgical procedures without clip placement. Hypertensive rats were confirmed with elevation of systolic BP ≥ 30 mmHg in comparison to the preclipping after 4 weeks of operation.

### 2.4. Electroacupuncture

**Figure 1:**
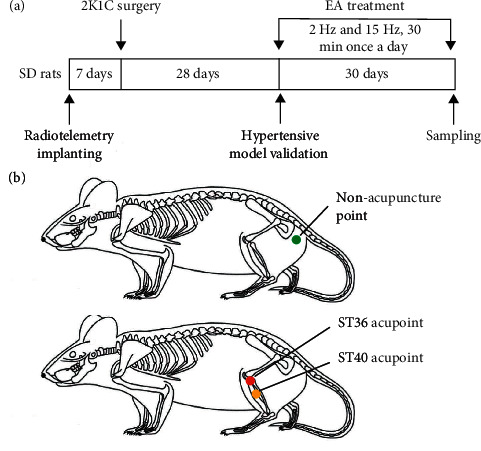
Diagram of the experimental procedures and the locations of the ST36 and ST40 acupoints or nonacupuncture point in a rat.

Four weeks after 2K1C or sham surgery as indicated in Figure 1, the EA treatment was performed in the immobilized conscious rats as previously described [21]. The acupoints of Zusanli (ST36) and Fenglong (ST40) were identified bilaterally using the standard acupoint map for rat in Experimental Acupuncture [22]. ST36 in a rat is located 5 mm lateral and distal to the anterior tubercle of the tibia, and ST40 is located at the midway between the ossa cruris and posterior margin of the fibula [21, 23]. Needles (0.16 mm diameter) for acupuncture were inserted perpendicularly at ST36 and ST40 in a depth of 2-3 mm bilaterally. Then an electrical stimulator (Hans-200A, Beijing Shengda Company, Beijing, China) was connected to the needles at the hind limbs and stimulated with alternate frequencies (2 Hz and 15 Hz) at certain intensity (≤4 mA) which evoked muscle twitches or movement of the paw for 30 min. EA treatment was performed at acupoints once a day for 30 days. The 2K1C + SEA group received EA stimulation out of acupoints, which are located at the junction between the tail and buttock, and was treated with the same electrical stimulation for 30 min every day. Needle insertion was applied in the sham control group and 2K1C group at ST36 and ST40 acupoints for 30 min every day but without electrical stimulation during the observation period. All therapeutic steps were operated by the same researcher following strict procedures.

### 2.5. Assessment of Changes in Sympathetic Activity

Power spectral analysis of BP variability was performed to assess the changes in sympathetic nerve activity as previously described [20]. Arterial BP recordings collected from the radiotelemetry system at a sampling rate of 500 Hz and 2 h of continuous recordings for each rat were analyzed using MATLAB software (MathWorks, Natick, MA, United States). The frequency domains were measured as follows: low frequency (LF, 0.2–0.8 Hz, measure of sympathetic tone), high frequency (HF, 0.8–2.5 Hz, measure of cardiac parasympathetic and potential respiration), Total Power (TP, 0.2–2.5 Hz), LF/HF (measure of sympathetic/parasympathetic balance), and LF/TP. The measures of autonomic tension were calculated.

In addition, BP responses to ganglionic blockade were performed to access the resting sympathetic vasomotor tone. At the end of the telemetry study, hexamethonium hydrochloride (30 mg/kg, iv) was administrated. The maximal change of MAP, which was considered as the index of sympathetic activity, was detected in rats [24]. The plasma levels of norepinephrine (NE) and epinephrine (*E*) were quantified to examine the sympathetic activity of rats by conducting the enzyme linked immunosorbent assay (ELISA, Rocky Mountain Diagnostics, Colorado Springs, CO, United States).

### 2.6. Evaluation Spontaneous Baroreflex Sensitivity

Spontaneous baroreflex sensitivity was evaluated using the sequence method as previously described [21]. The raw data of arterial pressure were collected from the radiotelemetry system as mentioned above and were analyzed with HemoLab software (Harald Stauss, Iowa City, IA). Individual baroreflex sequence which was defined as a sequence of at least four heart beats in which both systolic arterial pressure and pulse interval either simultaneously increased or decreased was plotted and subjected to linear regression (*r* threshold was set at 0.8). The baroreflex gain was calculated from the slope of the linear regression lines and expressed as up-sequence gain (in ms/mmHg), down-sequence gain (in ms/mmHg), and overall baroreflex gain (in ms/mmHg).

### 2.7. PVN Microinjection

The microinjection experiment was carried out after 30-day EA stimulating. Rats were anesthetized using 4% isoflurane for induction and 2-3% isoflurane exposure for maintenance. The left femoral artery and vein were cannulated with polyethylene catheters for the continuous direct BP recording and intravenous administration. In addition, the renal nerve was isolated and placed on a pair of silver electrodes after the left kidney undergoing retroperitoneal flank incision. Then the arterial catheter was connected to a pressure transducer attached to an amplifier (FE221, AD Instruments, Bella Vista, NSW, Australia). The nerve electrodes were attached to a differential AC amplifier (MODEL 1700, A-M Systems, Sequim, WA). The arterial BP and renal sympathetic nerve activity (RSNA) were recorded synchronously using a PowerLab data acquisition system (AD Instruments, Bella Vista, NSW, Australia).

PVN microinjection was conducted in accordance with reported procedure [18]. The rat was placed in a stereotaxic frame after being anesthetized with 3% isoflurane inhalation. After exposing the brain, a glass microinjection pipette (tip size 30–50 *μ*m) connecting with the microinjecting system was accessed to the PVN. The stereotaxic coordinates for PVN were 1.8 mm caudal from bregma, 0.4-0.5 mm lateral to midline, and 7.2–7.6 mm ventral to the dura in accordance with the rat atlas of Paxinos and Watson. The microinjections were performed bilaterally and each side microinjection was in a volume of 50 nL maintaining a period of 4 to 6 s. The body temperature of rat was maintained in the range from 36.5°C to 37.5°C by using a heating pad. After 30-day treatment, methylene blue dye (50 nL) was used for histological identification of microinjection site. Then the rats were euthanized with an overdose anesthetizing (sodium pentobarbital, 200 mg/kg, i.p.). Then the brain tissue was collected quickly. The data of rats were excluded from analysis if the microinjections were outside the boundary of the PVN.

### 2.8. PVN Tissue Microdissection and Real-Time PCR

The rats were euthanized with an overdose of sodium pentobarbital (200 mg/kg, i.p.), and the brain tissues were collected and cut into 500 *μ*m coronal sections. PVN tissues were dissected by punch-out technique as previously described [20], and pieces of cerebral cortex from the same section were also obtained. Total RNA from PVN and cerebral cortex was isolated in accordance with the procedure of the RNeasy kit (Qiagen, Valencia, CA, United States). Following reverse transcription of total RNA, the mRNA quantifications for NPY, NPY Y1R, and NPY Y5R were performed through TaqMan real-time PCR method as previously described [21]. TaqMan PCR probes for rat NPY(Rn01410145_m1), NPY Y1R (Rn02769337_s1), NPY Y5R (Rn02089867_s1), and 18S rRNA (Rn03928990_g1) were purchased from Thermo Fisher Scientific (Carlsbad, CA, United States). Applied Biosystems PRISM 7500 sequence detection system was used to detect the PCR reactions. Quantitative analysis of target mRNA expression was performed with a comparative cycle of threshold (CT) fluorescence technology. The expression level of the target genes was normalized by the expression of the 18S rRNA. The arbitrary units of target mRNA were expressed as the ratio of the target mRNA concentration to the 18S rRNA of the same sample.

### 2.9. Western Blot

PVN tissues were dissected out and homogenized in an ice-cold lysing buffer. Following determining the concentration of protein for each lysate, the proteins were loaded and separated by running in a SDS-PAGE gel, followed by electrophoretically transferring the protein to the nitrocellulose membranes (Millipore, Billerica, MA, United States). After being blocked overnight at 4°C using blocking buffer, the membrane was incubated with the primary antibodies at 4°C overnight, including mouse monoclonal anti-NPY (1 : 1000 dilution; #sc-133080, Santa Cruz Biotechnology), mouse monoclonal anti-NPY1-R (1 : 1000 dilution; #sc-393192, Santa Cruz Biotechnology), or NPY5-R (1 : 1000 dilution; #sc-137167, Santa Cruz Biotechnology). Then the membrane was incubated with conjugated secondary antibody at room temperature for 1 h and developed in the electrochemiluminescence (ECL). The images were collected from the film and identified by the Quantity One Software (Bio-Rad, Hercules, CA). The target protein levels were expressed as relative values against the *β*-actin level on the same gel.

### 2.10. Statistical Analysis

All data were expressed as means ± SEM. Statistical analysis was carried out using a software program (SigmaStat 4.0, Systat Software Inc., CA). Comparisons between experimental groups were performed with ANOVA followed by a Newman-Keuls test. Statistical significance was set as *P* < 0.05.

## 3. Results

### 3.1. Electroacupuncture Markedly Reduced MAP in 2K1C Rats

**Figure 2:**
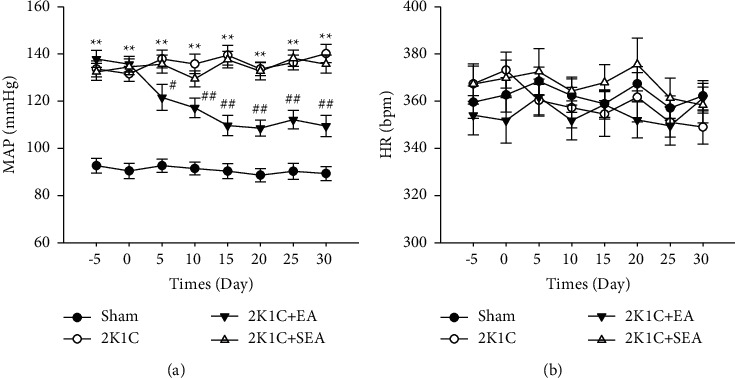
Depressor effect of electroacupuncture on 2K1C rats. (a) Time course of MAP. (b) Time course of HR. Data were expressed as mean ± SE; *n* = 6; ^*∗*^*P* < 0.05 and ^*∗∗*^*P* < 0.01 versus the SHAM group; ^#^*P* < 0.05 and ^##^*P* < 0.01 versus the 2K1C group.

The MAP and HR were measured for sham, 2K1C, 2K1C + EA, and 2K1C + SEA groups by radiotelemetry during the acupuncture treatment. As shown in Figure 2(a), the constriction of renal artery in rats by 4 weeks increased MAP and resulted in sustained hypertension. After 30 days of daily EA treatment at ST36 and ST40 acupoints, the MAP of 2K1C + EA group was significantly reduced from days 5 to 30 compared with the value of 2K1C group (*P* < 0.01). The MAP in 2K1C + SEA group which was subjected to nonacupoint stimulation was not significantly different from that in 2K1C group. Moreover, no notable changes in HR were observed among all the experimental groups (Figure 2(b)).

### 3.2. Electroacupuncture Significantly Attenuated Sympathetic Activities in 2K1C Rats

**Figure 3:**
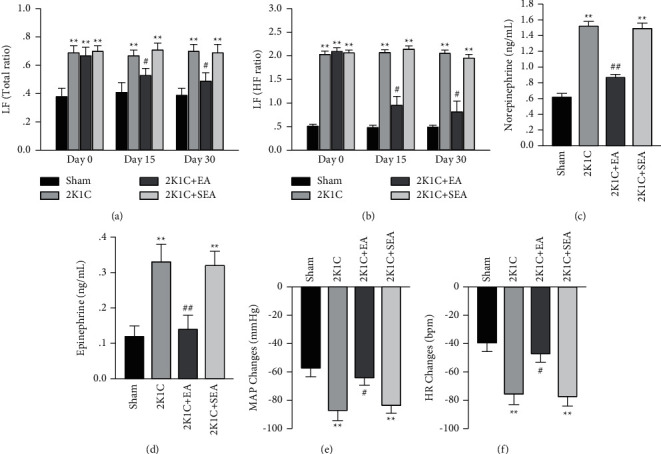
Sympathetic suppression effect of electroacupuncture in 2K1C group. (a) Changes of LF/Total ratios. (b) Changes of LF/HF ratios. (c) Plasma norepinephrine content. (d) Plasma epinephrine content. (e) The peak changed in MAP to hexamethonium. (f) The peak changed in HR to hexamethonium. Data were expressed as mean ± SE; *n* = 6; ^*∗*^*P* < 0.05 and ^*∗∗*^*P* < 0.01 versus the sham group; ^#^*P* < 0.05 and ^##^*P* < 0.01 versus the 2K1C group.

Spectral analysis of BP variability was applied in rats chronically implanted with telemetry to assess autonomic function. As shown in Figures 3(a) and 3(b), 2K1C rats had an increased low frequency BP variability, which exhibited remarkable and continuous rise in LF/Total and LF/HF ratios compared with sham rats. 30-day EA treatment in 2K1C rats markedly reversed the LF/Total and LF/HF ratios compared with sham operations in 2K1C group.

Plasma norepinephrine and epinephrine levels showed obvious increase in 2K1C rats compared to the sham group. After 30-day EA treatment, it was observed that the plasma norepinephrine and epinephrine levels were reduced significantly in 2K1C rats (Figures 3(c) and 3(d)).

Resting sympathetic vasomotor tone was accessed by depressor responses to ganglionic blockade. The peak decreasing in MAP and HR to hexamethonium was obviously greater in 2K1C group compared with sham group, and the 2K1C + EA group displayed significant improvement in depressor response of hexamethonium compared with 2K1C rats (Figures 3(e) and 3(f)).

### 3.3. Electroacupuncture Improved Baroreflex Sensitivity in 2K1C Rats

**Figure 4:**
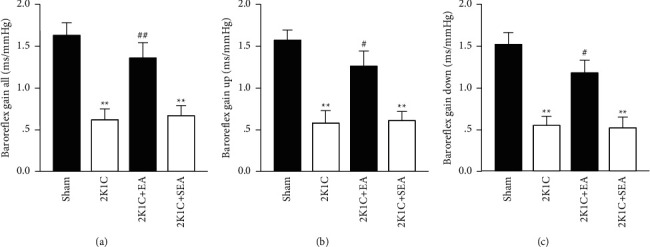
Electroacupuncture improved the spontaneous baroreflex gain in 2K1C rats. Spontaneous baroreflex analysis with overall baroreflex gain (up- and down-sequences) (a), up-sequence gain (b), and down-sequence gain (c) (in ms/mmHg). Data were expressed as mean ± SE; *n* = 6; ^*∗*^*P* < 0.05 and ^*∗∗*^*P* < 0.01 versus the sham group; ^#^*P* < 0.05 and ^##^*P* < 0.01 versus the 2K1C group.

Spontaneous baroreflex sensitivity was monitored consciously by the sequence method. 2K1C rats exhibited a significantly lower baroreflex gain (total, up-sequence, and down-sequence) compared to sham-operated rats. 30-day EA treatment in 2K1C rats significantly improved the impaired baroreflex function (Figure 4).

### 3.4. Electroacupuncture Increased NPY Expression in PVN of 2K1C Rats

**Figure 5:**
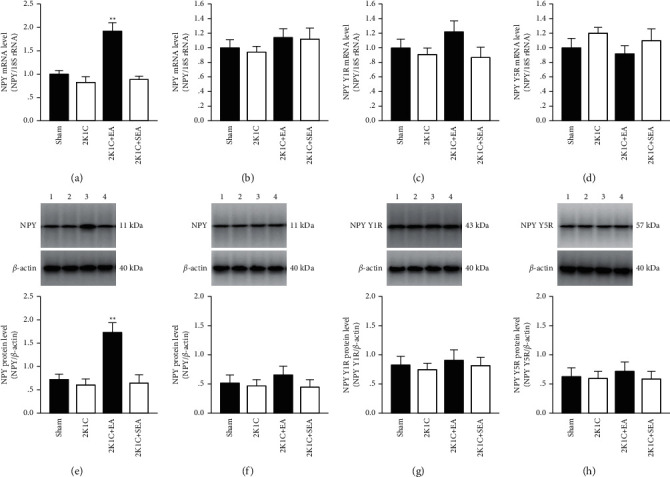
Electroacupuncture increases NPY expression in PVN of 2K1C rats. (a) NPY mRNA quantification within the PVN in each group. (b) NPY mRNA levels in the cerebral cortex from the same brain sections used for PVN assessment in each group. (c) NPY Y1R mRNA quantification within the PVN in each group. (d) NPY Y5R mRNA quantification within the PVN in each group. (e) NPY protein amounts within the PVN in each group. (f) NPY protein amounts in the cerebral cortex. (g) NPY Y1R protein amounts within the PVN in each group. (h) NPY Y5R protein amounts within the PVN in each group. Data were expressed as mean ± SE; *n* = 6; ^*∗*^*P* < 0.05 and ^*∗∗*^*P* < 0.01 versus the sham group; ^#^*P* < 0.05 and ^##^*P* < 0.01 versus the 2K1C group.

The expression of NPY system in the PVN of rats was examined by both real-time PCR and Western blots. As shown in Figure 5(a), NPY mRNA level in the PVN from the 2K1C + EA group was significantly elevated compared with sham group, 2K1C group, and 2K1C + SEA group. Consistent with increased mRNA, NPY protein levels were also markedly higher in the PVN of 2K1C + EA group compared with sham group, 2K1C group, and 2K1C + SEA group (Figure 5(e)). There were no remarkable alterations in NPY mRNA or protein level in the cerebral cortex from the same brain sections used for PVN assessment among all the experimental groups (Figures 5(b) and 5(f)). In addition, the protein and mRNA expressions of NPY Y1R and Y5R in the PVN also showed insignificant difference among all the experimental groups (Figures 5(c), 5(d), 5(g), and 5(h)).

### 3.5. Electroacupuncture Enhanced the Responses of PVN Microinjection with Neuropeptide Y (NPY) Y1R Antagonist BIBO3304

**Figure 6:**
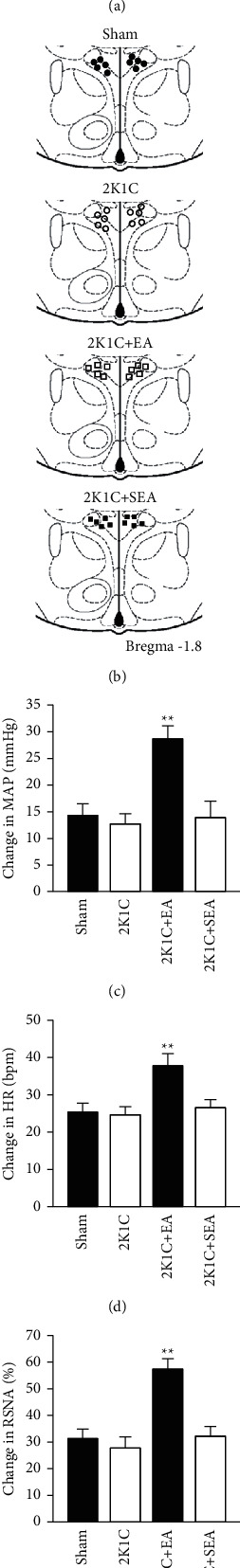
Electroacupuncture enhanced the responses of PVN microinjection with neuropeptide Y (NPY) Y1R antagonist BIBO3304. (a) Representative tracings showing AP, HR, and RSNA changes induced by microinjection BIBO3304 (1 mM in 50 nl) into the PVN of rats in all the experimental groups. (b) Histological placement for PVN microinjection sites. (c) Grouped data showing the peak alteration of MAP after PVN microinjection of BIBO3304 (1 mM in 50 nl). (d) Grouped data showing the peak alteration of HR after PVN microinjection of BIBO3304 (1 mM in 50 nl). (e) Grouped data showing the peak alteration of RSNA after PVN microinjection of BIBO3304 (1 mM in 50 nl). Data were expressed as mean ± SE; *n* = 5–7; ^*∗*^*P* < 0.05 and ^*∗∗*^*P* < 0.01 versus the sham group; ^#^*P* < 0.05 and ^##^*P* < 0.01 versus the 2K1C group.

To detect endogenous NPY tone from the PVN, specific NPY Y1R antagonist BIBO3304 was applied and bilaterally microinjected into PVN at the end of the 30-day operation. As shown in Figure 6(a), bilateral microinjection of BIBO3304 (1 mM in 50 nl) into the PVN increased the MAP, HR, and RSNA in all investigated groups. The peak alteration of MAP, HR, and RSNA caused by BIBO3304 in 2K1C + EA rats was distinctly greater than those in sham group, 2K1C group, and 2K1C + SEA groups (Figures 6(c), 6(d), and 6(e)). Microinjection of the same volume of vehicles (aCSF, 50 nl) in PVN of 2K1C + EA rats did not affect MAP, HR, and RSNA.  Figure 6(b) shows the histological placement of PVN microinjection sites in the rats of investigated groups. It failed to induce a significant alteration on MAP, HR, and RSNA by microinjecting BIBO3304 out of the PVN area (data not shown).

## 4. Discussion

The major findings of this experiment demonstrate that (1) EA intervention effectively reduces arterial BP, (2) EA treatment attenuates increased blood pressure in 2K1C rats associated with a reduction in markers of sympathetic vasomotor activity, (3) EA stimulating remarkably improves baroreflex sensitivity in 2K1C rats, and (4) EA treatment increases the NPY mRNA and protein expression in the PVN and enhances the responses of PVN microinjection with NPY Y1R antagonist BIBO3304 in 2K1C hypertension rats. These data suggest that the NPY system in PVN was involved in the antihypertensive and sympathetic suppression effect of EA treatment on hypertension rats.

Acupuncture, a traditional Chinese medicine treatment, has been applied for thousands of years for treating clinical conditions, including cardiovascular dysfunctions [1, 2]. Acupoint is the location of acupuncture stimulation, which is pathophysiologically associated with the conditions of internal organs and systemic functions according to traditional meridians theory [25, 26]. In this study, ST36 and ST40 acupoints were chosen for the following reasons: (1) based on traditional acupuncture theory, both ST36 and ST40 are the key acupoints in Stomach Meridian, which are clinically chosen for treating hypertension and prehypertension patients; (2) both ST36 and ST40 acupoints overly a dermatome of the peroneal nerves and the effects induced by EA at ST36 and ST40 in Chinese medicine practice are similar to the effects induced by peripheral neuromodulation. Numerous animal and clinical studies indicate that EA at Zusanli (ST36) alone, or in combination with other acupoints, has ameliorating and modulatory effects on hypertension [12, 27–31]. As a recommended nonpharmacological therapeutic method, EA at Zusanli exhibits effective results. However, the central mechanisms of EA treatment are still unclear. Consistent with these previous studies, we also confirmed that EA at ST36 and ST40 for 4 weeks significantly reduced the high BP in 2K1C hypertensive rats. What is noteworthy is the fact that insignificant alteration was observed in arterial BP after 4-week EA intervention at the nonacupoint in 2K1C hypertensive rats. The results observed indicate that the antihypertensive effects of EA treatment are acupoint-related more than the electrical stimulation on muscles.

It is well recognized that the sympathetic overdrive plays a pivotal role in the pathogenesis of hypertension and progression of organ damage [7]. Targeting the sympathetic activation directly therefore is considered as an effective approach on improving the hypertension and preventing cardiovascular events [8, 9]. Several studies have demonstrated that 2K1C renovascular hypertension was characterized by elevated sympathetic nerve activity [32, 33]. To explore the potential role of sympathetic activity in the beneficial effects of EA treatment, power spectral analysis of systolic blood pressure, plasma catecholamines, and depressor response to ganglion blockade were assessed to evaluate sympathetic tone. Our study showed that EA treatment markedly reversed the increased LF/Total and LF/HF ratios, reduced the plasma norepinephrine and epinephrine levels, and improved the greater depressor responses to acute ganglionic blockade in 2K1C hypertensive rats. These results suggest that EA treatments exert their antihypertensive effects at least in part by reducing the enhanced sympathetic activity.

The PVN is a unique and vital region in the brain which integrates and responds to a variety of neural and humoral signals regulating sympathetic tone. The preautonomic neurons of PVN innervate the sympathetic preganglionic neurons in the spinal cord via both direct and indirect projections, which is a primary origin of excitatory drive to the spinal sympathetic outflow [7, 8]. Increasing evidence indicates that the increased PVN neuronal excitability mediates elevated sympathetic drive in multiple hypertensive animal models [34, 35]. It has also been demonstrated that endogenous NPY was a potent inhibitory neuromodulator within PVN, which inhibits presympathetic neurons excitability and sympathetic tone [18, 36]. We therefore examined if EA treatment would elicit the PVN NPY release in hypertensive rats and thus blunt the enhanced sympathetic activity. Our findings verify that EA stimulating at acupoints significantly increases the expression of NPY mRNA and protein in PVN and enhances the responses of PVN microinjection with NPY Y1R antagonist in 2K1C hypertensive rats. These data suggest that EA-induced reduction of sympathetic activity in hypertension may be through enhancing the NPY sympathoinhibitory mechanism originating within the PVN.

NPY is a potently endogenous neuropeptide in the hypothalamus, which has been implicated in both orexigenic and sympathoinhibitory effects [16, 17]. The PVN was identified as a specific brain region where NPY suppresses sympathetic nerve activity, which was densely innervated by NPY-containing axons of arcuate nucleus origin, and forms functional inhibitory synapses on PVN presympathetic neurons [18, 19, 37]. This action is mediated mainly through Y1 and Y5 receptors, which are expressed in the PVN [17]. In this study, the expressions of NPY Y1R and Y5R were detected in the PVN tissues of investigated groups, and the results indicate that the mRNA and protein levels of NPY Y1R and Y5R exhibited no significant difference among groups. These observations may imply that the Y1 and Y5 receptors of NPY are not involved in the sympathoinhibitory effects of EA treatment. We also found that the NPY level in the cerebral cortex was not altered in rats of investigated groups. Thus, the increased NPY expression in PVN of hypertensive rats might be related to EA application, which is probably the underlying mechanism for antihypertensive effect of EA.

One concern is the possible afferent pathways of acupuncture action on modulation of brain neuronal activity. From traditional Chinese medicine, acupoints are specific somatic areas which make a somatointernal organ connection through meridians [25]. Histological studies reveal that acupoints are located in regions with high density nerve distribution [26]. It has been evidenced that stimulation at specific acupoint could modulate somatic nerves that project to most of brain areas, which primarily regulate autonomic outflow and cardiovascular function [6, 38]. Several studies also demonstrated that removal of the nerves innervating acupoints using capsaicin and selective neurectomies eliminated the therapeutic effect of acupuncture [39, 40]. Meanwhile, the specific pathway by which sensory signals inspired by acupuncture transit the PVN to activate outflow regulation activities needs further study.

In summary, our data revealed that antihypertensive and sympathoinhibitory actions of acupuncture depend on the upregulated NPY system in PVN of hypertensive rats. These experimental results provide evidences to support the beneficial effects of EA intervention on hypertension. Therefore, acupuncture at acupoints could potentially be a novel clinically therapeutic method against hypertension.

## Data Availability

The data used to support the findings of this study are available from the corresponding author upon request.
